# New wine in old skins: Scopoletin biosynthesis in cotton

**DOI:** 10.1093/plphys/kiae527

**Published:** 2024-10-04

**Authors:** Henryk Straube

**Affiliations:** Assistant Features Editor, Plant Physiology, American Society of Plant Biologists; Faculty of Science, Department of Plant and Environmental Sciences, Section for Plant Biochemistry, University of Copenhagen, 1871 Frederiksberg C, Copenhagen, Denmark

A major factor for crop productivity is pathogen pressure, and human-made climate change affects this pathogen pressure. Tropical areas will likely experience little to no growth in crop production, partly due to the rise in plant diseases (Chaloner et al. 2021).

The dominant fiber crop is cotton (*Gossypium hirsutum*), with a worldwide production of 25 million metric tons (Hamawand et al. 2016). Farmers grow cotton predominantly in tropic and subtropic regions. Cotton yield is threatened by vascular wilt disease caused by the pathogenic soil-born fungus *Verticillium dahliae*. *Verticillium* is inaccessible during its growth, its spores persist over long times in the soil, and genetic resistance is rare (Klosterman et al. 2009). The most effective countermeasure is to use gaseous pesticides on the soil, reducing the load of *Verticillium* spores in the soil (Klosterman et al. 2009).

Plants evolved many mechanisms to counteract fungal infections. One defense mechanism is synthesizing specialized metabolites, known as phytoalexins, that have antimicrobial, growth-inhibiting effects to fend off pathogens in response to an infection (Ahuja et al. 2012). One class of phytoalexins consists of coumarin derivatives, synthesized from phenylpropanoid precursors (Fig.). Scopoletin, a blue fluorescent coumarin derivative, was first described in the late 1800s, with its biosynthesis pathway uncovered a century later (Gnonlonfin et al. 2012). Although the exact transcriptional regulation of phytoalexin biosynthesis genes remains unclear, the rapid production of these compounds following pathogen infection suggests a complex network of transcriptional regulators at play.

**Figure.**
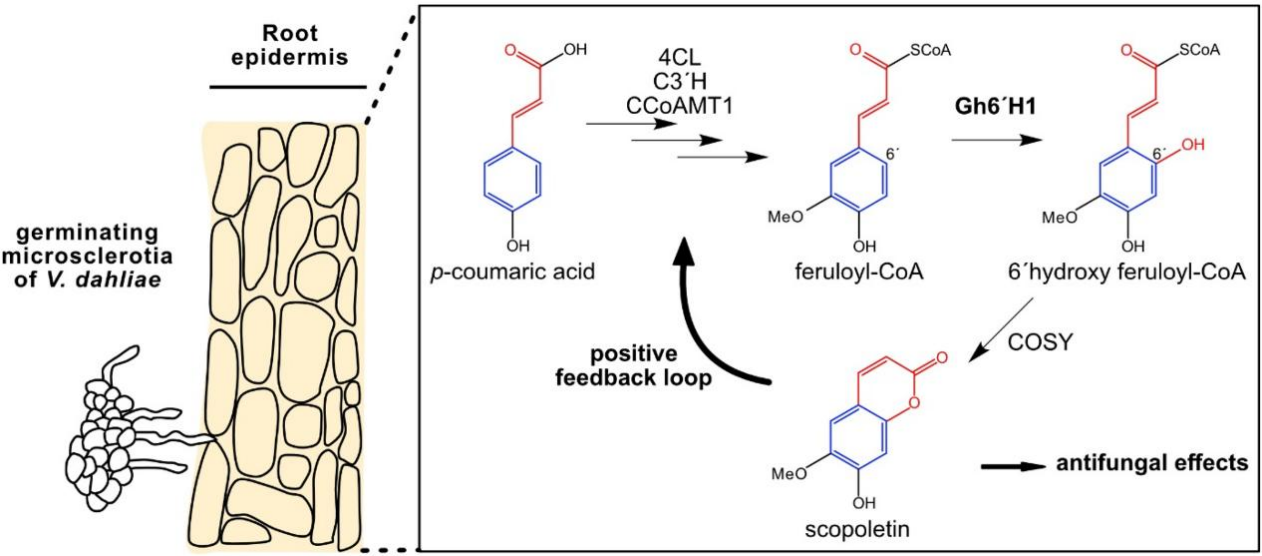
Biosynthesis and regulation of scopoletin in cotton. Scopoletin is synthesized through multiple enzymatic steps in the phenylpropanoid pathway in response to infection by *V. dahliae*. Scopoletin has antifungal properties and creates a positive feedback loop, increasing the transcription of its biosynthesis genes. Moieties forming the benzol ring in the scopoletin are depicted in blue, whereas moieties forming the 2-pyrone ring are drawn in red. COSY, coumarin synthase.

Scopoletin displays strong antibacterial and especially antifungal properties, inhibiting germ tube elongation and conidium germination of several fungi (Gnonlonfin et al. 2012). A recent study showed that engineering the coumarin pathway increases stress resilience in several plants (Beesley et al. 2023).

In this issue of *Plant Physiology*, Gao et al. (2024) studied the role of scopoletin in the interaction of cotton and *V. dahliae*. The authors uncovered the involvement of a 2-oxoglutarate-dependent dioxygenase from cotton, FERULOYL-CoA 6-HYDROXYLASE 1 (GhF6'H1), in scopoletin biosynthesis, and cotton defense response to *V. dahliae*.

Scopoletin treatment of *V. dahliae* mycelium resulted in morphological changes like shriveled surfaces of sporocarps, abnormal cytoplasmatic organelles, thinner cell walls, and distorted septa. Testing the leakage of nucleic acids and proteins from mycelium, the authors revealed that following the observed phenotypical differences, scopoletin treatment leads to increased leakage of macromolecules from the mycelium. An infection of cotton plants with *V. dahliae* resulted in increased concentrations of scopoletin, and treating leaves with 250 *μ*M scopoletin made plants less susceptible to *V. dahliae*. Surprisingly, exogenous scopoletin treatment led to systemic increases in endogenous scopoletin levels not only in the leaves but also in the roots and stems.

Since scopoletin proved effective against *V. dahliae*, Gao and colleagues analyzed the transcript levels of several homologous genes in cotton potentially involved in scopoletin biosynthesis, following *V. dahliae* infection. Several of the candidate genes were upregulated, including a homolog of the well-characterized Arabidopsis (*Arabidopsis thaliana*) gene *FERULOYL-CoA 6-HYDROXYLASE 1* (Kai et al. 2008; Beesley et al. 2023) named *Gh*F6**'*H1*. The transcript of *Gh*F6**'*H1* was upregulated after fungal infection and when cotton plants were treated with methyl jasmonate, salicylic acid, and, most surprisingly, scopoletin itself.

Using *Tobacco rattle virus* (TRV) to perform virus-induced gene silencing (VIGS), the researchers observed that decreased transcript amounts of *Gh*F6**'*H1* reduced concentrations of scopoletin and consistently increased susceptibility of cotton plants to *V. dahlia*. Infection of plants containing less transcript of *Gh*F6**'*H1* with *V. dahliae* resulted in higher amounts of scopoletin, although the increase was less than in the plants treated with the VIGS control.

Plants carrying the TRV:*GhF6'H1* showed reduced transcript levels of homologous genes potentially involved in scopoletin biosynthesis, including *4CL1* (*CoA LIGASE 1*) and *CCoAOMT1* (*CoA O-METHYLTRANSFERASE 1*), while *C3'H* (*P-COUMARATE 3′-HYDROXYLASE*) remained unaffected. The transcripts of *4CL1* and *CCoAOMT1* showed a smaller increase after infection in plants carrying the control VIGS or TRV:*GhF6′H1*.

To explore the biotechnological potential of GhF6′H1, the authors created stable transgenic cotton plants, overexpressing *Gh*F6′H1**. These plants displayed increased transcript abundances of *Gh*F6′H1** and increased concentrations of scopoletin. As in previous publications (Beesley et al. 2023), the *Gh*F6′H1* overexpression lines were more resistant to fungal infection with *V. dahliae**. Complementing the treatment of plants with exogenous scopoletin and the VIGS experiments, plants overexpressing *Gh*F6′H1** had increased transcript amounts of *4CL1*, *CCoAOMT1*, and *C3′H*. These results were further confirmed repeating the overexpressor experiments in Arabidopsis, leading to similar results.

In summary, Gao et al. (2024) characterized the homolog of the well-characterized enzyme *FERULOYL-CoA 6-HYDROXYLASE 1* in cotton and its involvement in resistance against *V. dahlia*. Interestingly, their results suggest that scopoletin exerts positive feedback on its biosynthetic pathway across multiple plant species (Fig.). These findings contribute to the ongoing discussion on the transcriptional regulation of specialized metabolite biosynthesis pathways and offer a valuable example (Kliebenstein 2023; Li et al. 2024). Future research should explore the mechanisms behind plant perception of small molecules, which will undoubtedly lead to many exciting discoveries.

## Data Availability

There are no new data associated with this article.
